# A tai chi/qigong intervention for older adults living with HIV: a study protocol of an exploratory clinical trial

**DOI:** 10.1186/s13063-020-04728-x

**Published:** 2020-09-22

**Authors:** Gladys E. Ibañez, Kristopher Fennie, Linda Larkey, Nan Hu, Angel B. Algarin, Chelsea Valdivia, Helen Lavretsky

**Affiliations:** 1 Department of Epidemiology, Florida International University, 11200 SW 8th St AHC5, Miami, FL 33199 USA; 2 Division of Natural Sciences, New College of Florida, 5800 Bay Shore Road, Sarasota, FL 34243 USA; 3 College of Nursing and Health Innovation, Arizona State University, 550 N 3rd St, Phoenix, AZ 85004 USA; 4 Department of Biostatistics, Florida International University, 11200 SW 8th St AHC5, Miami, FL 33199 USA; 5 Division of Public Health, Family and Preventive Medicine, University of Utah School of Medicine, Salt Lake City, Utah 84132 USA; 6 Division of Infectious Diseases and Global Public Health, University of California San Diego, La Jolla, CA 92093 USA; 7 Department of Psychiatry, Semel Institute for Neuroscience and Human Behavior, The University of California, Los Angeles (UCLA), 760 Westwood Plaza, Los Angeles, CA 90095 USA

**Keywords:** Qigong, Tai chi, HIV, Older adults, Intervention, Mind-body

## Abstract

**Background:**

Almost half of people living with HIV (PLWH) in the USA are over 50 years of age; this is expected to increase to 70% by 2020. Yet, few interventions exist for older PLWH that address psychological and physical symptoms combined, both prevalent in this population. There is a need to find innovative and accessible interventions that can help older PLWH to manage their symptoms. Mind-body interventions, like tai chi/qigong (TCQ), improve both physical and psychological health. TCQ is a series of slow, low-impact meditative movements that integrates breathwork, meditation, and stances.

**Methods:**

The present study is an exploratory clinical trial that will evaluate the acceptability and feasibility of a 12-week, small group TCQ intervention (*n* = 24), a sham qigong control condition (*n* = 24), and a standard of care control condition (*n* = 24) for older people living with HIV/AIDS. It will also explore any preliminary associations between the TCQ intervention and symptom alleviation. Participants will be recruited from community-based health and social services organizations in Miami, FL, and randomized to one of the 3 conditions.

**Discussion:**

We will assess feasibility and acceptability through questionnaires and adherence to TCQ. We will assess preliminary associations with symptoms such as depression, anxiety, social support, chronic HIV-related fatigue, and clinical outcomes. These will be described through proportions, means, and changes over time through graphing techniques. Outcomes will be assessed at baseline, at post-intervention, and at 3 months follow-up. These preliminary analyses also will provide information necessary to estimate effect size and power needed for a larger clinical trial.

**Trial registration:**

ClinicalTrials.gov NCT03840525. Registered on 16 July 2018.

## Introduction

The prevalence of HIV in the USA is highest among older adults (45+ years old), with people over 50 representing 45% of those who are living with HIV in the USA [1]. During 2012–2016, the largest percentage increase in prevalence rates (55.7%) was among persons 65 years and older [1]. Older PLWH also report more psychological and physical symptoms than their younger counterparts. A recent study found older PLWH reported more frequent agitation, depression, anxiety, apathy, irritability, and nighttime sleep disturbances than younger PLWH [2]. Older PLWH are 2–3 times more likely to suffer from depression than the general population [3, 4]. Older PLWH also report lower levels of physical ability than younger PLWH [5]. HIV decreases bone mineral density and increases osteoporosis, making older PLWH at an increased risk of fall-related injuries [6, 7]. Interventions are needed to address both the psychological and physical symptoms related to aging with HIV.

Mind-body interventions, like tai chi/qigong (TCQ), improve both physical and psychological health. TCQ is a slow, meditative movement exercise that uses breathwork, meditative focus, and fluid body movements to promote health. TCQ is considered “meditative movement” [8] and exercise for the mind as well as the body [9]. In a review of randomized clinical trials (RCTs) of TCQ and mental disorders, it was found that TCQ RCTs reported significant improvement in depression, stress, and anxiety, and had significant positive effects on mood and psychological well-being [10]. TCQ is known to also ameliorate physical health conditions such as cardiovascular conditions, hypertension, asthma, cancer [11–13], and diabetes [14]; all are common comorbidities found in PLWH. Moreover, this year, there have been several reviews and meta-analyses supporting the health benefits of TCQ including better immunological functioning [15], higher quality of life and less depression [16], anxiety [17], less hypertension [18], and general health promotion [19].

Despite growing evidence of the efficacy of TCQ to alleviate various chronic conditions, only a handful of intervention studies exist examining TCQ among PLWH. One RCT with PLWH found that TCQ was linked to quality of life and improved functional capacity [20], less emotion-focused coping [21], and lower HIV-related psychological distress [22]. In sum, there is a gap in knowledge regarding the range of health benefits of TCQ for PLWH, as well as the acceptability and feasibility of a TCQ intervention with older PLWH. The purpose of this study is to describe the protocol for an exploratory clinical trial of a TCQ intervention with older PLWH. The primary outcome of the clinical trial is the acceptability and feasibility of a TCQ intervention, and the secondary outcomes are to explore the effects of TCQ on psychological and physical symptoms among older PLWH.

## Materials and methods

### Overview

The Gentle Empowering Movement (GEM) Study will measure the acceptability and feasibility of the TCQ intervention compared to a sham qigong (SQG) group, which is intended to control for physical activity and serve as an attention-control condition. There will also be a standard of care control group, which will not receive any services from the study. Both control groups will be offered the intervention after study completion.

This clinical trial was approved by the institutional review boards (IRB) at Florida International University and Arizona State University. It is funded by the National Center for Complementary Integrative Health (NCCIH) and registered on www.clinicaltrials.gov [NCT03840525].

#### Trial status

The clinical trial enrolled its first participant on January 6, 2020, and recruitment was ongoing until COVID-19. The study is currently suspended due to COVID-19 and will begin again once it is safe to reopen. The first cohort of intervention participants began February 7, 2020. This study protocol is version 5.0, created November 20, 2019. The anticipated completion date is December 2021.

### Study site

The trial will be conducted at a partnering clinic, which is registered as a federally qualified health center focusing on minority populations in the inner city. The center’s target population consists primarily of Hispanics 56%, Haitians and African Americans 39%, Whites, and others 5%. The majority (86.9%) earns an annual income at or below the federal poverty level.

### Theoretical framework for the intervention

**Fig. 1:**
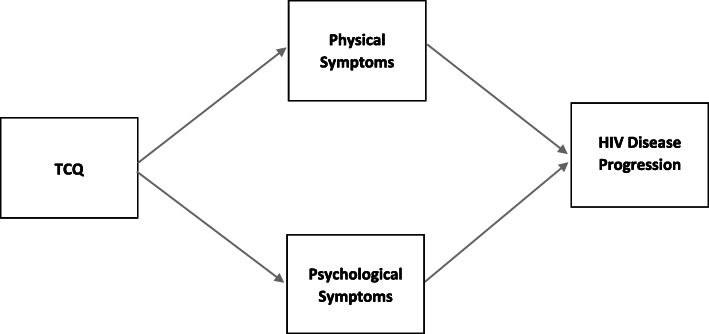
Theoretical framework adapted from Spirduso et al. (2008) exercise-cognition model

Researchers adapted Spirduso et al.’s exercise-cognition model [23] to fit the target outcomes and population (see Fig. 1). Our model focuses on the relationship between exercise (i.e., TCQ) and disease, using physical and psychological mediators. The theoretical framework links the components of TCQ to physical and psychological symptoms that in turn are expected to influence clinical outcomes. The secondary outcomes of the present clinical trial are these physical and psychological symptoms.

### Training instructors and DVD production

The study co-investigator, a certified senior instructor of TCQ, trained intervention facilitators. The training lasted approximately 25 h: 8 h conducted via Zoom (v5.1.2; San Jose, CA) videoconferencing, followed by another 16–17 h of training in person in order to be certified. The trainer also trained the SQG control group facilitators. This training lasted 4–8 h including mock teaching. The senior TCQ instructor also met with the facilitators on a regular basis (weekly for the first month, then monthly) via phone and/or Zoom to discuss any issues that arise during the intervention. There is also an accompanying video that all facilitators and participants will have access to; participants use this video to help with their qigong home practice. This video was professionally produced by a video production company. A Spanish version of the video used for home practice sessions was also produced, and therefore, we have both English and Spanish versions of the TCQ and the SQG exercises for these study conditions, respectively, for participants to use during their home practice. These videos were recorded using the TCQ and SQG facilitators. In addition to the video, each participant receives a booklet displaying all the movements learned in the group.

### Study design

#### Recruitment and screening

This is a 3-arm randomized controlled exploratory clinical trial. Participants (*n* = 72) are recruited via clinic staff referrals, recruitment flyers, and/or clinic presentations. They are screened in person or over the phone by the research staff to confirm eligibility and scheduled for a baseline assessment. Participants are eligible if they are 50 years or older, living with HIV, able to provide consent, and willing and able to participate for the length of the intervention period (12 weeks). Participants are asked to provide the year in which they were first diagnosed with HIV by a healthcare professional, and the ability to provide consent will be assessed using the GAIN cognitive impairment scale, whereas the individual must score ≤ 10 points to be deemed cognitively able [24]. Additionally, participants are provided with a thorough explanation of the study procedures and expected time commitment before they asked if they are willing and able to participate. Participants are excluded from the study if they report being unable to stand for 10-min segments (i.e., wheelchair or walker bound), or if the participant has substantial (i.e., regular weekly practice for more than 3 months in the past 12 months) experience with mind-body practices (tai chi, qigong, or yoga), they will be excluded because the control group may be contaminated by prior experience.

#### The TCQ intervention

The TCQ intervention is standardized and manualized and has a formal training program for instructors (www.instituteofintegralqigongandtaichi.org). The intervention content includes a series of repeated and easy to learn movements that are also the forms known to be linked to health benefits [25]. The TCQ intervention content includes three main components: (1) the three “intentful” corrections, (2) four movements based on the vitality method of qigong, and (3) six movements based on tai chi. The three “intentful” corrections form the basis for all subsequent movements and focuses on proper posture, breathwork, and clarity of mind as participants do the movements. Movements are taught standing and sitting on chairs for those who are unable to stand for of the entire class time (see Table 1 for list of movements). The steps and duration of the sham qigong group are in Table 2. The rationale of the sham is to serve as an attention-control group since the main research interest is in the meditative aspect of the qigong group. We attempt to ensure non-effectiveness of the sham qigong control group by the following: sham instructors are trained to keep the group as rote as possible, we conduct booster trainings as needed, and instructors are video recorded as they teach and intervention fidelity is assessed by a senior investigator using these videos.

**Table 1:** Main components of the TCQ intervention

Week 1	The three intentful corrections
	Aligning, opening, and concluding tai chi easy sequences
Weeks 1–4	Vitality method—flowing motion
	Vitality method—right and left bending of spine
	Vitality method—front and back bending of spine
	Vitality method—reaching upward, stretching outward
Weeks 5–12	Spontaneous qigong
	Channeling energy
	Tai chi movement 1—harmonizing yin and yang
	Tai chi movement 2—brush knee send qi (chi)
	Tai chi movement 3—cutting the path to clarity
	Tai chi movement 4—watching clouds pass
	Tai chi movement 5—gathering heaven and earth

**Table 2:** Main components of the SQG intervention

Weeks 1–12	Warm-up and cool down	Stretching neck
		Rolling shoulders
		Shrugging shoulders
		Body twist
	Main movements	Hug your head
		Lunges
		Side stretch
		Reaching for the stars
		Dance step
		Swinging arms
		Hanging circles
		Overhead stretch
		Floor stretch
	Alternative movements	Marching
		The swim
		Dusting the room
		The swing
		Reverse swim

#### The sham qigong (SQG) and the standard of care control condition

The SQG is a physical activity- and attention-control group that uses similar types and intensity of movements that are part of the TCQ intervention, but without the meditative state and breath focus that is present in the TCQ intervention. The third arm will be a standard of care control group and will receive no services. After trial completion, participants in both control conditions will be offered the intervention. See Table 3 for the characteristics of all 3 conditions.

**Table 3:** Descriptive characteristics of the TCQ intervention and control conditions

	TCQ intervention	SQG control	Standard of care group
Intervention features	• Low-impact physical activity • Focus on breath • Meditative state • Incidental social support	• Low-impact physical activity with same/similar movements as TCQ intervention • Incidental social support	• No exercise or activity program
Dose/frequency	• 12 weeks, 1/week 60 min class • 2× per week in first 2 weeks • Approx. 2 ½ h home practice/week	• 12 weeks, 1/week 60 min class • 2× per week in first 2 weeks • Approx. 2 ½ h home practice/week	• n/a
Controls for	• Unique focus; breath and meditative state	• Low-impact physical activity	• n/a

### Criteria for discontinuing allocated interventions

If a review of adverse events shows significant harms or if there is any breach of confidentiality, the study will be stopped, and a review of existing procedures will be conducted.

### Adherence/incentive strategies

Based on some of the elements of social cognitive theory, we will use role-modeling, social support, and intrinsic reward to help participants adhere to the intervention. These strategies are the following: (1) the instructor will communicate with each participant when they come to the session to reinforce self-efficacy, (2) a monthly 1-page newsletter will be given to all participants with success stories and wellness information, and (3) a “buddy” system will be encouraged to promote participation. In addition, the research coordinator will make weekly phone calls as a reminder of upcoming sessions and to help promote the at-home practice. There will also be monetary incentives for participating in each of the assessments. Thirty dollars will be provided for the baseline and 2-week follow-up assessments, respectively, and $50 for the 3-month follow-up assessment. However, if participants attend at least 80% of the intervention sessions, they will earn an additional $20 at the 2-week follow-up assessment, that is, $50. In addition, we will provide transportation assistance via UberHealth© for those participants who need and request it.

### Sample size and randomization

**Fig. 2:**
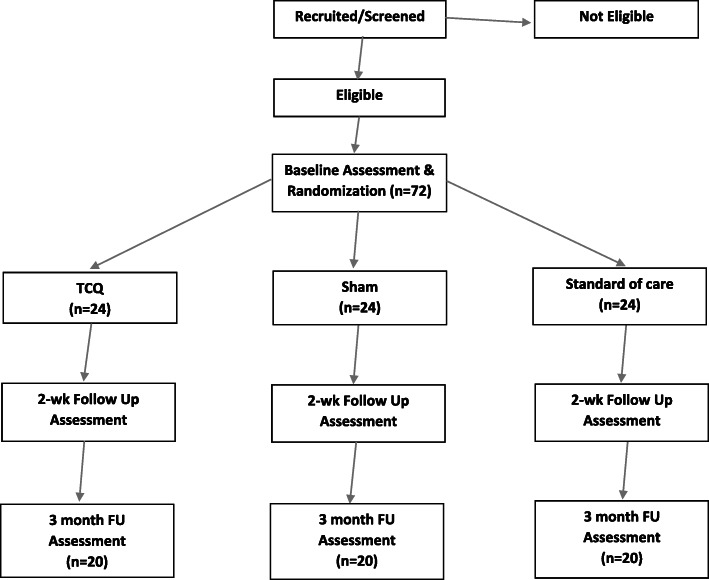
Study design flowchart

This is a pilot study that is not designed to test efficacy and as such is not powered for hypothesis testing. However, given a sample of 72 individuals, and an acceptability and feasibility proportion of 80%, the precision of the measurement would be 95% confidence interval of 68–89%, and by intervention group 61–92%. We used a random block design to randomize participants into one of three treatment groups (TCQ, SQG, standard of care) in three equal blocks of 24, whereby each block would have 24 participants randomized into the three treatment groups. The rationale for three blocks was logistical, so that assignment into the three treatment groups would be more even. The procedure for randomization involved creating a SAS® dataset that contained the structure necessary for the design, including an initial subject counter (1–72) and three treatment groups, nested in blocks. We then used the Plan Procedure in SAS® using a seed to randomly assign each subject counter a treatment group within a block. The resulting list was imported into Excel, and a sequential participant identification was added. The randomization assignment list was housed with the study manager in a locked file cabinet. Neither the interviewer nor the statistician had access to the list. Immediately prior to or after an enrolled person’s baseline interview, the study manager assigned the participant to the next available treatment condition, in sequential order (Fig. 2).

### Blinding

For logistical reasons, the study will be partially blinded. Those randomized to the TCQ and SQG conditions will be blinded to which group they were assigned, whereas the standard of care control group will know they are assigned to the control condition based on format and content of the condition. Data collectors and analysts will be blinded to group assignment. Participants will also be blinded. Success of the blinding will be assessed by asking participants to guess which condition they attended during the 3-month follow-up interview. The study research coordinator and principal investigator are unblinded throughout the study.

### Assessments

Once eligibility is determined, participants are scheduled for a baseline assessment. All assessments are conducted in a private research office space. Bilingual research staff will be trained to conduct the survey in order to collect the data, if needed. Participants are consented and administered the baseline measures using computer-assisted personal interviewing procedures with REDCap® software. Baseline assessments last approximately 45 min and include demographic items, psychological and physical symptoms measures, adherence items, and viral load. At 2-week follow-up and 3-month follow-up, the same measures are administered (see Table 4 for a description of all measures). Most measures have a Spanish version, but for those that do not, a back-translation procedure was used to translate measures [37].

**Table 4:** All measures and time of administration

Quantitative measures	No. of items/psychometrics	Screen	BL	Weekly/during intervention	2-week FU	3-month FU
**Eligibility** [24]						
Age, HIV status (self-report/lab test)		X				
Any mind-body practice in past 12 months		X				
GAIN cognitive impairment	6 items/α = .87	X				
Can stand up for 10 min		X				
**Demographics and covariates** [26–30]			X			
HIV-related stigma	6 items/α = .75					
Social support scale	12 items/α = .94					
Drug use (DAST)	20 items/α = .92					
Alcohol (AUDIT)	10 items/α = .83					
**Psychological symptoms** [31, 32]						
CES-D scale			X		X	X
Depression, Anxiety and Stress Scale (DASS-21)	21 items/α = .86					
**Physical symptoms** [33, 34]						
The Revised Sign and Symptom Checklist for HIV	45 items/α = .71–.91		X		X	X
HIV-related Fatigue Scale (MAF)	16 items/α = 0.88		X		X	X
**Clinical** [35]						
5-item medication adherence questions MMAS-8	8 items/α = .75		X		X	X
Viral load (lab results)			X		X	X
**Feasibility**			X	X	X	
**Acceptability**				X	X	
**Feasibility of implementation in clinic setting** (interventionists reporting)					X	
**Intervention fidelity** [36]						
Meditative Movement Inventory	9 items/α = .86–.90			X	X	
Intervention Fidelity Checklist (for senior trainer)				X		

### Acceptability measures

Acceptability is measured by administering a brief satisfaction survey to participants after each intervention session and comparing them to the control conditions. We collect acceptability metrics such as attendance, information on home practice sessions including how often they practiced and how long each practice session was, and satisfaction with the DVD and practice sessions. Acceptability of the home practice sessions and adherence strategies is collected during weekly calls to participants. Based on Sekhon et al.’s [38] work, there are specific benchmarks to determine the acceptability of the intervention (see Table 5).

**Table 5:** Acceptability and feasibility definitions and benchmarks

	Definition	Benchmark
**Aspects of acceptability being measured (adapted from Sekhon et al.** [38]**)**		
Burden	Discontinuation/non-attendance	< 20% of TCQ and the SQG condition will drop out at 3 months follow-up.
Ethical consequences	Any side effects with intervention	< 10% of participants will report any physical soreness or difficulties in doing TCQ.
Experience	Participant’s experience and satisfaction	> 80% TCQ participants will perceive intervention positively.
		> 80% TCQ participants will rate it very satisfactory or satisfactory.
Affective attitudes	Participants attitudes toward the intervention	> 80% of participant in TCQ condition will report positive attitudes about it (very good/good).
Opportunity costs	Adherence and participation	Participants in both TCQ and SQG conditions will attend at least 75% of all sessions.
		Participants in the TCQ condition will adhere to home practice at least 75% of the time expected (i.e., 112 out of 150 min/week).
Intentions	Willingness to participate	> 80% TCQ participants would be willing to participate again.
		> 80% TCQ participants intend to continue qigong practice.
**Aspects of feasibility being measured (adapted from Bowen et al.** [39]**)**		
Demand	Likelihood that intervention will be used	> 80% of TCQ participants will report any TCQ practice in the past week.
Implementation	Can the intervention be implemented in a setting, often a real-world setting	Trainers will execute intervention > 80% of the time.
		> 80% of trainers will rate resources needed to implement as very good/good.
		> 80% of trainers will report positive effects on population.
Practicality	Extent to which an intervention can be delivered given the limited resources	> 80% of trainers will report participant’s ability to do the intervention as very good/good.
Integration	Extent to which the intervention fits the system	> 80% of trainers will perceive intervention fits the clinic infrastructure as very good/good.
		> 80% of trainers perceive the sustainability of the intervention as very good/good.

### Feasibility measures

Feasibility metrics include percentage of those who are eligible, consented, and randomized. The percentage of who completed the 2-week and 3-month follow-up assessments are calculated. The percentage of who did the home practice sessions are collected via weekly call. The intervention facilitators complete a survey at the end of each intervention cohort (12 weeks duration) to assess whether the intervention was feasible to implement in a clinic setting (see Table 5).

### Intervention fidelity

All intervention and control group sessions are video recorded and stored on a secured server accessible only to the principal investigator and co-investigators. Our senior trainer views 10–20% of these video recordings and rates them on intervention fidelity using an intervention fidelity measure developed for the study.

### Confidentiality

All data collected is identified only by a participant identification number to maintain confidentiality. All paper-based records are also kept in a locked file cabinet in the research office which was also locked nightly. In addition, data collected via laptops are encrypted and stored on a secured cloud server.

### Data analysis plan

Data obtained through REDCap® will be imported into SAS® version 9.4. Acceptability and feasibility data will be collected through paper-based questionnaires and logs and tracked through RedCAP® and imported into SAS®. Paper-based questionnaires and logs will be reviewed by a research staff for completeness and consistency. Paper-based questionnaires will be double entered to ensure accuracy of data entry. Prior to analysis, data will be cleaned by examining frequencies, means, medians, and ranges to identify logical errors. Instruments will be coded according to their respective scoring instructions. Unless specified by the instrument, missing items will be imputed based on averaging values within the instrument for a given individual, if no more than 15% of items are missing. Otherwise, that instrument will be set to missing for the individual.

Because this is an exploratory clinical trial, analyses will be primarily descriptive; hypotheses will not be assessed through statistical significance, in accordance with recommendations for analysis of pilot studies [40]. Feasibility, acceptability, and clinical outcomes will be described through proportions, means, and changes over time, and 95% confidence intervals (CI) will be calculated to estimate precision. Regarding clinical outcomes, researchers will focus on estimating treatment effect sizes and respective 95% CI, which will help to determine the sample size needed for a larger scale RCT. Baseline demographic and clinical characteristics, including psychological and physical symptoms, will be summarized by treatment condition and compared for even distribution of characteristics to assess effectiveness of the randomization.

To address acceptability among participants, data will be used from the satisfaction survey which will be administered weekly during the intervention and at post-intervention. Individual items from the survey such as percent sessions attended, frequency of home practice sessions, average length of home practice sessions, and questions on opinion of the intervention sessions, home practice sessions, and video will be plotted over time to examine variability of acceptability measures over time. Next, participant’s average score over the intervention period will be averaged. These averages, as well as the items from the post-intervention satisfaction survey, will be categorized into acceptable or not acceptable. Benchmarks used to determine acceptability and feasibility were predetermined in collaboration with the sponsoring agency (see Table 5). From these data, we will determine the proportion of participants who rate the intervention as acceptable. To estimate precision, 95% CI will be calculated using Wilson’s score method, which uses asymptotic variance and is appropriate for small sample sizes [41]. The formula is as follows: (2*np* + *z*^2^ ± √(*z*^2^ + 4*npq*))/2(*n* + *z*^2^). We also will conduct the above analysis on both control conditions. Certain questions on the satisfaction survey will differ with the control condition, but we anticipate similar levels of acceptability among the three conditions. Lastly, we plan to repeat these descriptive analyses among three separate strata: gender, current drug users, and age groups (50–65 and 65+), assuming variation within strata. We anticipate that there could be differences in acceptability among these different groups.

Clinical, psychological, and physical outcomes will be assessed at baseline, at post-intervention, and at 3 months follow-up. The Depression, Anxiety and Stress Scale-21 (DASS-21) instrument will be used to assess depressive symptoms, anxiety-related symptoms, and stress. To assess physical symptoms, the Revised Sign and Symptom Checklist for HIV and the HIV-related Fatigue Scale will be used [33, 34]. To assess clinical outcomes, adherence to antiretroviral medicine will be measured using a 5-item medication adherence questionnaire. Adherence will be set at 95%. Viral load at baseline and at 3 months (if available) will also be collected.

Gauging preliminary efficacy is helpful in determining effect sizes; thus, while we will not be testing for statistical significance, we will conduct the below described models and examine confidence intervals, in order to help estimate effect size. We will describe outcome data using univariate and bivariate statistics. For categorical outcomes, we will look at frequencies at each time point. For dichotomous outcomes, a repeated measures logistic regression will be completed using the Genmod and Glimmix Procedures that will include group, time, and a group by time interaction, followed by multiple comparisons of the interaction term to determine changes over time within each time condition as well as differences among the groups at post-intervention and 3-month follow-up. A 95% CI will be used to help determine effect size. Univariate analyses will be conducted for continuous outcomes, including mean, median, range, and standard deviation. Distributions will be assessed for normality. Univariate analyses will be conducted on the outcomes stratified by gender, current drug use, and age groups. Then, a repeated measures linear regression will be conducted, using the Mixed Procedure, with a group, time, and group by time interaction, followed by multiple comparisons. The Power Procedure in SAS® and GPower® will be used to estimate the sample size needed to test differences in outcomes among the three treatment conditions in a future RCT.

### Interim analyses

In addition, interim analyses will be conducted. A data safety monitoring board (DSMB) was established for this clinical trial. It will meet every 6 months to review the progress of the clinical trial, and determine if discontinuation or continuation is justified based on the interim report. The interim analyses will include information on accrual, baseline characteristics, and other general information on study status. This report will also contain data on study outcomes, safety, and serious adverse events (AEs).

### Oversight and monitoring

This is a feasibility study being conducted at one site, and therefore, there is no coordinating center. However, the data management team, consisting of the principal investigator, co-investigators, consultant, and research coordinator, meet monthly to discuss study progress. In addition, the principal investigator also meets with staff on a weekly basis to review study progress.

The DSMB consists of at least 3 members. Two members constitute a quorum. The members have been approved by the NCCIH and will include at least one member with human subject research monitoring expertise, at least one member with relevant disease expertise (such as an M.D. or equivalent), at least one member with expertise in the intervention or observation technique under study, and a Ph.D.-level biostatistician. The DSMB responsibilities include the following: review the research protocol, study materials, and all proposed revisions; evaluate the progress of the study; consider factors that may impact the safety of the study participants; and make recommendations to the NCCIH, the Grantee Institution, the principal investigator (PI), and the IRB concerning continuation, termination, or other modifications of the study. Members of the DSMB have no financial, scientific, or other conflict of interest with the study. Current or past (within 3 years) collaborators, including any individual involved in the design, conduct, or analysis of the study; associates; and direct reports of the PI are not eligible to serve on the IMC. Meetings are attended, when appropriate, by the study principal investigators (PI(s)) and members of the study team. A formal report containing the recommendations for continuation or modifications of the study, prepared by the DSMB, will be sent to the study PI(s). It is the responsibility of the study PI(s) to distribute this report to all co-investigators and the NCCIH and to the IRB associated with the study. Each DSMB report should conclude with a recommendation to continue or to terminate the study.

All research staff will also be trained on the definitions of SAEs and AEs and its reporting procedures. Staff will notify the PI immediately after the occurrence of an SAE or AE. SAEs will be reported to NIH and to the IRB within 24 h, with a written report within 48 h. All AEs that occur during the study will be documented and reported by the investigators to NIH and the FIU IRB at the time of their continuing annual reviews. The principal investigator and research coordinator will review study materials for quality assurance periodically throughout the year. Protocol amendments will be submitted to the IRB and to NCCIH for prior approval before implementation. Protocol amendments will also be reported to the DSMB during scheduled meetings. Based on previous literature, we anticipate little to no study-related AEs except for some minor muscle soreness [42]. If minor soreness is reported, we will report the AE to the IRB and funder during our interim review, and we will discuss the event during our weekly meetings with the instructors and how to address this issue in order to minimize future AEs.

### Dissemination of findings

The investigators will share research findings with the public and the scientific community through presentations at conferences and as requested by the NIH. Papers will be published in both peer-reviewed journals and reports to the NIH.

### Data sharing plan

After the completion of the study, data will be made available to researchers upon review and approval of the submitted protocols by the research team. Researchers will need to sign an agreement to protect the confidentiality of the data and obtain prior approval from their respective IRBs, the Florida International University IRB, and the NIH. The full protocol is available via the principal investigator.

## Discussion

The GEM Study is an exploratory clinical trial that will pilot test a TCQ intervention with older PLWH. If found acceptable to this population, TCQ programs could be an effective tool in managing both physical and psychological symptoms experienced by those living and aging with HIV. It will be an approach that can complement any medication regimen and is low impact enough that older PLWH could participate.

The study may pose potential limitations. One limitation may be the potential for diffusion of information. Participants recruited from similar sites may know one another and may share their experience during the trial period. To assist with minimizing this potential limitation, we will adjust for study site location. Another limitation may be generalizability as this trial is only among PLWH aged 50+ years of age. If the trial is found to be effective, it may not be generalizable to younger PLWH.

One of the strengths of the study is that the TCQ intervention will be compared to a similar control (SQG) condition to determine whether it is the physical aspect or the meditative aspect of qigong that is related to symptom management. Another unique aspect of this study is the home practice. Although the home practice has been included in previous studies using the intervention, this is the first time that we offer the video via streaming in addition to the DVD, and it is the first time that the video home practice will be developed in Spanish. For example, results may demonstrate whether older PLWH will be amenable to seeing the TCQ home practice on their smartphones.

There is still much research to be done with qigong interventions. If found acceptable and feasible, a larger clinical trial will be proposed to further examine the efficacy of qigong for managing physical and psychological symptoms, as well as other aspects of disease progression such as immune functioning and cognitive impairment. In sum, qigong has the potential to cross racial, gender, and fitness levels; serves as a complementary activity to any HIV treatment regimen; and may be well-suited for a population experiencing numerous physical and psychological symptoms such as older PLWH.

## Data Availability

Not applicable.

## Abbreviations

PLWH: People living with HIV
TCQ: Tai chi/qigong
SQG: Sham qigong
RCT: Randomized clinical trial
GEM: Gentle Empowering Movement
NCCIH: National Center for Complementary Integrative Health
SAE: Serious adverse event
AE: Adverse event
DSMB: Data safety monitoring board
NIH: National Institutes of Health
